# Can Persistent Blue Light Exposure Cause a Molecular Shift in the Vitreous of Rodents?

**DOI:** 10.1155/sci5/4875029

**Published:** 2026-05-23

**Authors:** Nagarajan Theruveethi, Manjunath B. Joshi, Manna Valiathan, Radhika R. P., Shama Prasada Kabekkodu, Shailaja Bhat

**Affiliations:** ^1^ Department of Optometry, Manipal College of Health Professions, Manipal Academy of Higher Education, Manipal, 576104, Karnataka, India, manipal.edu; ^2^ Department of Ageing Research, Manipal School of Life Sciences, Manipal Academy of Higher Education, Manipal, 576104, Karnataka, India, manipal.edu; ^3^ Department of Pathology, Kasturba Medical College, Manipal Academy of Higher Education, Manipal, 576104, Karnataka, India, manipal.edu; ^4^ Department of Cell and Molecular Biology, Manipal School of Life Sciences, Manipal Academy of Higher Education, Manipal, 576104, Karnataka, India, manipal.edu; ^5^ Department of Ophthalmology, Kasturba Medical College, Manipal Academy of Higher Education, Manipal, 576104, Karnataka, India, manipal.edu

**Keywords:** amino acids, blue light blocking lenses, mass spectrometry, metabolites, short wavelength

## Abstract

This experimental rodent study aimed to explore changes in the vitreous metabolome caused by prolonged chronic environmental blue light stress and to elucidate the underlying mechanisms. Four separate groups of control (NC), blue light (LE), two blue blocking lenses (blue light–blocking lenses (BBLs), BL + Crizal Prevencia (CP) and BL+Duravision Blue protect (DP) and 8‐week‐old albino male Wistar rats were used in this experiment. Animals were subjected to blue LED light, with and without BBLs, for 90 days under a 12:12‐h light:dark cycle and constant illumination at 450–500 lux. The control animals were maintained in standard laboratory conditions. Postexposure, the vitreous fluid (VF) was aspirated, stored at −20°C, and processed for LC‐MS. We observed significant variation in amino acid (AA) abundance across four groups: normal control (NC), light exposure (LE), light exposure with Duravision Blue (DB) and light exposure with CP lenses. Specifically, there was a significant difference in AA content between NC and LE [*F* (21, 178) = 4.667, *p* < 0.0001], between LE and CP [*F* (21, 181) = 7.971, *p* < 0.0001], between LE and DB [*F* (21, 179) = 13.06, *p* < 0.0001], between NC and CP [*F* (21, 184) = 11.46, *p* < 0.0001] and between all groups in general [*F* (21, 182) = 14.22, *p* < 0.0001]. Pathway‐level analysis with false discovery rate (FDR) correction identified significant enrichment in phenylalanine and tyrosine metabolism, valine/leucine/isoleucine degradation and beta‐alanine metabolism pathways. From these results, it can be inferred that BBLs and light exposure significantly influence the presence of AAs compared with NC and LE. Persistent exposure to cumulative blue light can alter vitreous metabolites, potentially affect micro‐ and macromolecular components of the VF and carry functional consequences for vitreoretinal health.

## 1. Introduction

The vitreous fluid (VF) that fills the posterior parts of the eye provides physical and chemical support to maintain intraocular physiology. This can potentially safeguard the lens and trabecular meshwork by converting retinal oxygen into ascorbate, thereby reducing oxidative stress in these critical ocular components [1–3]. Indoor LED lighting emits a blue diode covered by a yellow chromophore; these LED spectral characteristics become imbalanced due to a peak in the blue region, which produces high energy and can lead to light‐induced damage in the intraocular environment. There is a greater risk of blue light‐mediated alteration in the VF for shorter [28 days, 12:12 light:dark (LD)] and longer durations (90 days, LD) [4, 5]. These factors may impair VF metabolism and compromise visual system health [6].

Commercially available blue light–blocking lenses (BBLs) are designed to reduce exposure to hazardous blue light. These lenses use a reflective coating or absorption technology to effectively block blue light [7]. The BBLs have attracted the attention of consumers and eye care practitioners, highlighting the importance of protective measures to safeguard our eyes from harmful blue light exposure [8]. In vitro studies have examined the efficacy of BBLs postcumulative light exposure for shorter durations on vitreous metabolites and have demonstrated that BBLs partially restore several essential metabolites [4]. In vivo studies using coloured lenses protect against the phototoxicity of retina‐suppressing blue light [9].

Clinical studies indicate that BBLs do not alleviate visual symptoms of eye strain, convergence or accommodation [10] or significantly improve sleep and mood, though there is a partial improvement in sleep quality in individuals with sleep depressive syndrome [11]. It is essential to investigate the adaptive or maladaptive response system of the vitreous to chronic light exposure and how commercially available BBLs protect against prolonged exposure to these LEDs [10–13]. Although blue light can temporarily damage retinal and vitreous cells, there is currently mixed evidence suggesting that it can trigger photochemical reactions in vitreous cells, leading to the degeneration or alteration of biochemical compounds. We hypothesise that prolonged exposure to blue LED light may lead the visual system to develop a compensatory mechanism or worsen the intraocular environment by altering metabolite levels.

Although knowledge gaps exist regarding the impact of blue light on the vitreous, ongoing studies indicate that understanding the mechanisms underlying potential harm from cumulative exposure is important. It is crucial to safeguard the visual system from excessive exposure to blue light, especially from LED lights and digital devices. Using a metabolomics approach, our study aimed to assess the metabolomic profile of rat VF samples after 90 days of exposure to blue LED light. Additionally, we explored the effectiveness of BBLs in providing protection. To investigate this, we designed an experiment involving 90 days of exposure to 12:12 h dark:light (D:L) cycles of blue LED light and commercially available BBLs to evaluate the potential ameliorative effects of these treatments. This prolonged exposure paradigm models chronic environmental blue light exposure rather than acute phototoxic stress and may have secondary effects on circadian regulation.

## 2. Methodology

### 2.1. Ethical Statement

We received approval from Institutional Research and Institutional Animal Ethics at Kasturba Medical College, MAHE (IAEC‐KMC‐02/2017), and followed CPCSEA guidelines procedures. All procedures involving animals were conducted in accordance with the ARRIVE guidelines. The animals used in the study were procured from the Central Animal Research Facility (CARF), MAHE, Manipal.

### 2.2. Experimental Framework

In a standard laboratory setting, a group of Albino Wistar rats were given unrestricted access to food and divided into three experimental groups and one control group (*n* = 6). One group was exposed to blue light (L.E., *n* = 6), while the other two groups wore either Crizal Prevencia lenses (CP, *n* = 6) from Essilor in Charenton‐le‐Pont, France, or Duravision Blue lenses (DB, *n* = 6) from Carl Zeiss in Oberkochen, Germany. The light source, an ES‐EMBCF22 L‐A fitted with an InGaN‐series blue LED chip from Hsinchu, Taiwan, was placed on top of the cage, with a 50‐cm distance between the light source and the rats.

In the experiments, eight‐week‐old male rats were housed in pairs and separated only during light exposure periods. These rats were subjected to blue light exposure for 90 days, with a consistent lighting schedule of 12 h on and 12 h off and an illumination level of 450–500 lux. The rats in the blue light–blocking group were given lenses to filter out blue light. The control group of rats was maintained in standard laboratory lighting conditions without blue enrichment. As outlined, the animals were grouped based on light exposure into LE and BBL (CP and DB). The rats were housed individually in separate cages during exposure periods, with *L* = 100 cm, *W* = 70 cm, and *H* = 50 cm, and vertical curtains were used to maintain distinct light intensities and qualities for each rack.

To minimise confounding variables, all experimental groups were housed in the same animal facility under identical environmental conditions (22°C ± 2°C, 50 ± 10% humidity, standard bedding). Animals had ad libitum access to standard rodent chow and water, with feeding schedules and cage maintenance synchronised across groups and performed during the same circadian phase to reduce feeding‐related metabolic variation. All animals underwent a 1‐week acclimatisation period before light exposure initiation, and standardised handling protocols were employed by the same personnel across all groups to minimise handling‐related stress variation. Critically, all animals were euthanised and sampled at the same Zeitgeber time (ZT12, end of light phase) following the 90‐day exposure period, with sample collection order randomised across groups and consistent processing times (< 5 min from euthanasia to vitreous aspiration and freezing at −20°C). These measures were implemented to control for potential confounding effects of stress, feeding cycles, circadian phase and handling on metabolomic profiles.

Following a week of laboratory adaptation, we began light exposure at 9:00 p.m., with a total duration of 90 days for long exposure under a 12‐h dark and 12‐h light cycle. After exposure, all animals were euthanised with an intraperitoneal injection of a lethal dose of pentobarbital (i.p. 100 mg/kg Euthasol) and xylazine (10 mg/kg Proxylaz). Then, the eyes were enucleated using watchmaker forceps (number 5) and Sklar’s blunt enucleation scissors. Following eyeball removal, an aspiration technique using a 21‐gauge hollow needle was used to extract the vitreous, which was then preserved at −20°C for LC‐MS analysis.

### 2.3. BBLs

The following BBLs were used for the experiment: From 9:00 p.m. to 9:00 a.m., a blue LED at a wavelength of 400–490 nm with a brightness of 450–500 lux (Ee = Ev/km(*λ*) and Ee = 0.123 W/m^2^) was used to illuminate the eyes of each rat for 12 h. We used two types of BBL spectacle lenses with the following parameters.

**FIGURE 1:**
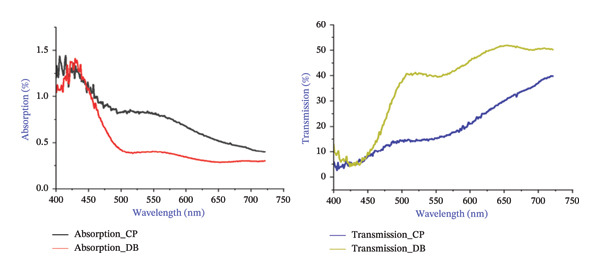
Spectral characteristics of blue light–blocking lenses. Demonstrates the spectral transmission and absorption properties of Crizal Prevencia (CP) and Duravision Blue (DB) lenses across the visible spectrum (380–720 nm). Relative intensity (arbitrary units), absorption (%) and transmission (%) measured using SHIMADZU IRSprit‐T Fourier transform infrared spectrophotometer with QATR‐S single reflection ATR accessory (measurement step size 5 nm).

BBL‐1 CP lenses: Essilor et al., 1.56, reflection 8.1%–19.8%, transmittances (*T*%) = 0.530, mean absorption = 0.779 and yellow chromophore index (YCI). BBL‐2 DB lenses: Carl et al., 1.5, yellow selectivity, transmittances (*T*%), mean absorption = 0.530 and YCI. The optical properties of visible light transmittances (*T*%) and range of wavelengths (380–720) were measured (measured step size = 5 nm and calculated step size = 5 nm) using a spectrophotometer (SHIMADZU, IRSprit‐T, Fourier transform infrared spectrophotometer, QATR‐S, Single reflection ATR accessory) (Figure 1).

### 2.4. Metabolomic Analysis of the Vitreous

Postexperiment, the VF was extracted from N.C., LE and BBLs (CP and DB) for LC‐MS analysis. The VFs were transferred to aliquoted tubes, and frozen methanol was added twice. The mixture was then centrifuged at 12,000 rpm for 15 min. The supernatant was collected, dried using a SpeedVac (SRF110P1‐115) and reconstituted in 1.0 mL of LC (liquid chromatography)‐matching solvent to ensure kiconsistency with internal standards. Finally, the samples were transferred to LC vials and analysed using LC‐MS with a temperature‐controlled autosampler.

**FIGURE 2:**
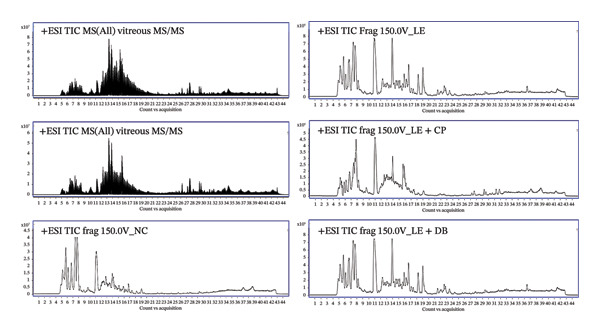
LC‐MS metabolomics workflow and data processing. Summary of standardisation methods for MS/MS data from all experimental groups. The peak chromatogram displays total ion current (TIC), encompassing TIC normalisation and internal standard (I.S.) normalisation for untargeted metabolomics. Data acquired from normal control (NC), light exposure (LE) and blue light–blocking lens groups (LE + CP and LE + DB).

For untargeted LC‐MS/MS metabolite analysis, an Agilent LC‐MS/MS system was used. Eight‐microlitre aliquot samples were injected into an Agilent 1290 LC system coupled to an ESI‐Q‐TOF MS/MS 6520 instrument (Agilent Technologies, Santa Clara, CA, USA). The HPLC column (Phenomenex, Torrance, CA, USA) was maintained at 25°C. Chromatographic separation took 20 min each, and the total analysis time per sample was 36 min. The MS/MS data were acquired, and the peaks were integrated using Agilent Mass Hunter software. Datasets for more than 80% of the samples presented in the group were accounted for and normalised with log10 transformation. Autoscaling was applied (computing z‐scores) before test statistics were calculated using Mass Profiler Pro (MPP). We reported the mass, RT, *M*/*Z* ratio and amino acid (AA) abundance of the animals in the light‐exposure control and experimental groups (Figure 2).

Metabolite identification was performed according to the metabolomics standards initiative (MSI) guidelines. Level 1 identification (confirmed with authentic standards) was achieved for major AAs, including glutamate, aspartate, phenylalanine, tyrosine, leucine, isoleucine, valine, glycine, serine, threonine, lysine and proline, analysed under identical chromatographic conditions. Level 2 identification (putatively annotated) was assigned to metabolites matched by accurate mass (< 5 ppm), retention time and MS/MS fragmentation patterns against the METLIN and HMDB databases, without authentic standard confirmation.

### 2.5. Statistical Analysis

Statistical analysis was performed using R Version 3.5.2 software. Principal components were analysed using Agilent MPP software (p/n G3835‐90021). The VF metabolomic data were analysed using metabolomics pathway analysis (MetPA) in MetaboAnalyst, Version 5.0. The abundance values of the identified metabolites were log_5_ transformed for subsequent analysis. Pearson’s correlation was used to assess correlations between metabolites across groups. A one‐way ANOVA was used to test significant differences in metabolite levels across groups.

For pathway enrichment analysis, false discovery rate (FDR) correction was applied using the Benjamini–Hochberg procedure in MetaboAnalyst 5.0. Both raw *p* values and FDR values are reported. Individual metabolite comparisons across groups were performed using one‐way ANOVA; nominal *p* values are reported, while biological interpretation focuses on metabolites within FDR‐corrected significant pathways.

### 2.6. Pathway Analysis

We analysed the *p* values of metabolites across four groups within a specific pathway. We aimed to determine the overall effect of multiple metabolites on pathway enrichment. To achieve this, we calculated the geometric mean, the *n*th root of the product of all *p* values. The value *n* represents the total number of metabolite groups (Table 1). To further analyse the pathway effect, we used MetaboAnalyst 5.0 and identified significant pathway enrichment values. These values are crucial for understanding whether a pathway is over‐ or underrepresented in the data and its observed impact.

**TABLE 1:** Metabolic pathway enrichment analysis with multiple testing correction.

Pathways	Total	Expected	Hits	Raw *p* value	FDR
Phenylalanine and tyrosine metabolism	28	0.465	3	0.00964	0.351
Ammonia recycling	32	0.531	3	0.014	0.351
Valine, leucine and isoleucine degradation	60	0.996	4	0.0143	0.351
Beta‐alanine metabolism	34	0.564	3	0.0165	0.351
Aspartate metabolism	35	0.581	3	0.0179	0.351
Glutathione metabolism	21	0.349	2	0.0453	0.74
Arginine and proline metabolism	53	0.88	3	0.0532	0.745
Methylhistidine metabolism	4	0.0664	1	0.0649	0.795
Urea cycle	29	0.481	2	0.081	0.842
Lysine degradation	30	0.498	2	0.0859	0.842

*Note:* Pathways were analysed using the MetaboAnalyst 5.0 pathway analysis module. ‘Total’ indicates the total number of metabolites in the pathway; ‘Expected’ represents expected hits based on random chance; ‘Hits’ shows the actual number of metabolites matched from our dataset; ‘Raw *p* value’ from the hypergeometric test; ‘FDR’ represents the false discovery rate calculated using the Benjamini–Hochberg procedure for multiple testing correction. Pathways are ranked by raw *p* value. FDR < 0.05 indicates pathways remaining significant after correction for multiple comparisons (Table 1).

Pathway analysis: we analysed the *p* values of metabolites across four groups within a specific pathway. We aimed to determine the overall effect of multiple metabolites on pathway enrichment. To achieve this, we calculated the geometric mean, the *n*th root of the product of all *p* values. The value ‘*n*’ represents the total number of metabolite groups (Table 1). To further analyse the pathway effect, we used MetaboAnalyst 5.0 and identified significant pathway enrichment values.

## 3. Results

In this study, we investigated the impacts of blue light exposure and BBLs on VF under light exposure (LE) conditions and compared them to those of age‐matched controls [normal controls (NCs)]. LC‐MS untargeted metabolomics analysis identified 33 biologically relevant metabolites in VFs, including some not reported in short‐term light‐exposure studies. The majority of AAs reported were confirmed with authentic standards (MSI Level 1), while pathway intermediates were putatively annotated (MSI Level 2) based on database matching. We utilised METLIN and HMDB (The Metabolomics Innovation Centre (TMIC), the Human Metabolome Database) databases to determine the biological relevance of their findings.

In this study, 22 metabolites were analysed across different groups. Only a few metabolites, such as phenylalanine and tyrosine, exhibited increased abundance in the LE and BBL groups. However, AAs such as threonine, catecholamines, glycine and serine were found to be dysregulated. Lysine and glutamate, on the other hand, remained relatively stable. Finally, increased glutamate metabolism was observed in the LE and BBL samples.

Pathway‐level analysis with FDR correction identified several significantly enriched metabolic pathways (Table 1), providing confidence that the observed metabolomic shifts reflect genuine biological perturbations rather than multiple testing artefacts. Pathway enrichment analysis (Table 1) revealed significant alterations in phenylalanine and tyrosine metabolism, valine/leucine/isoleucine degradation, beta‐alanine metabolism and glutathione metabolism. These pathways converge on oxidative stress responses, retinal neuronal metabolism and systemic metabolic homoeostasis, providing a mechanistic framework for understanding how chronic exposure to blue light affects vitreoretinal biochemistry.

Data analysis and trends: based on the visual analysis of the data points and their associated error bars: general comparison: across the majority of the 22 metabolites, the LE group (red) consistently shows lower abundance than the NC, CP and DB groups. This suggests a systemic downregulation of these metabolic pathways under low‐energy conditions. High‐abundance metabolites: compounds such as L‐isoleucine (131) and L‐asparagine (132) show the highest abundance levels, reaching near $10^8$ in the NC, CP and DB groups, while remaining significantly lower (around $10^7$) in the LE group. Low‐abundance metabolites: taurine (125) and L‐histidine (155) are among the least abundant metabolites in the LE group, often dipping below $10^5$, whereas they remain significantly higher in the other groups. Variability: the error bars indicate the variance within each group. The LE group often shows larger relative error bars at lower abundances (e.g., for taurine and citrulline), suggesting greater biological or technical variability at these levels. Group‐specific observations: LE vs. NC: the NC group is generally 2–5 times more abundant than the LE group for most metabolites. LE vs. CP: the CP group shows a trend similar to NC, with particularly high levels of branched‐chain AAs (valine and L‐isoleucine). LE vs. DB: the DB group shows the most pronounced differences in metabolites such as glutathione (307) and inositol 1,4,5‐trisphosphate (420), in which the LE group is significantly depleted.

Box plots show the distribution of AA abundance in VF from four groups. Statistical analysis: one‐way ANOVA with post hoc pairwise comparisons. Significance levels: NC vs. LE: *F* (21, 178) = 4.667, *p* < 0.0001; LE vs. CP: *F* (21, 181) = 7.971, *p* < 0.0001; LE vs. DB: *F* (21, 179) = 13.06, *p* < 0.0001; NC vs. CP: *F* (21, 184) = 11.46, *p* < 0.0001; overall comparison: *F* (21, 182) = 14.22, *p*  < 0.0001.

**FIGURE 3:**
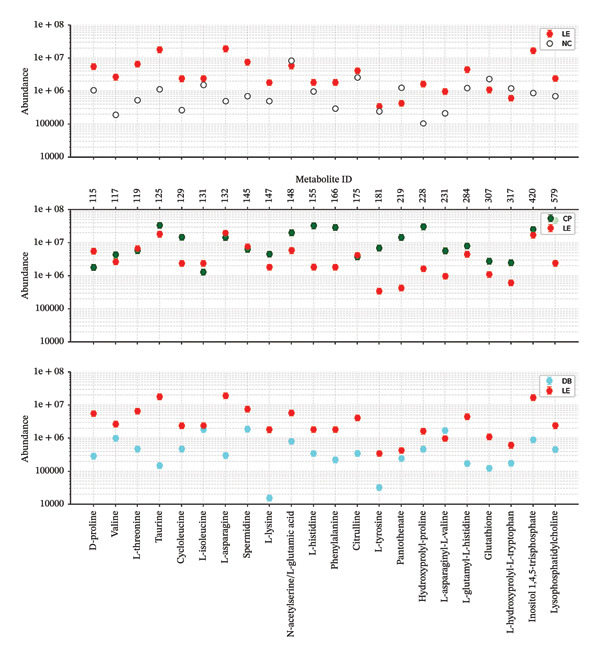
Amino acid abundance across experimental groups. Box plots showing distribution of amino acid abundance in vitreous fluid from four groups: normal control (NC), light exposure (LE), light exposure with DB lenses (LE + DB) and light exposure with CP lenses (LE + CP). Boxes represent interquartile range, horizontal lines indicate median and whiskers extend to 1.5× IQR.

One‐way ANOVA showed that there was a significant difference in the abundance of AAs among the four groups: the NC, LE, DB light exposure and CP lens light exposure groups (Figure 3). Specifically, there was a significant difference between NC and LE in AAs [*F* (21, 178) = 4.667, *p* < 0.0001], between LE and CP AAs [*F* (21, 181) = 7.971, *p* < 0.0001], between LE and DB AAs [*F* (21, 179) = 13.06, *p* < 0.0001], between NC and CP AAs [*F* (21, 184) = 11.46, *p* < 0.0001] and between all groups in general [*F* (21, 182) = 14.22, *p* < 0.0001]. The findings indicate that the presence of AAs and light exposure is significantly affected by BBLs compared with NC and LE.

## 4. Discussion

This study represents the first metabolomic analysis of VF following chronic (90 days) exposure to blue LED light, addressing a critical gap between acute phototoxicity studies and real‐world cumulative exposure scenarios. Unlike short‐term (28 days) exposure paradigms that primarily model acute stress responses, our 90‐day protocol, conducted under controlled 12:12 h light–dark cycles, simulates chronic environmental exposure to blue‐enriched indoor lighting while maintaining rudimentary circadian architecture.

**FIGURE 4:**
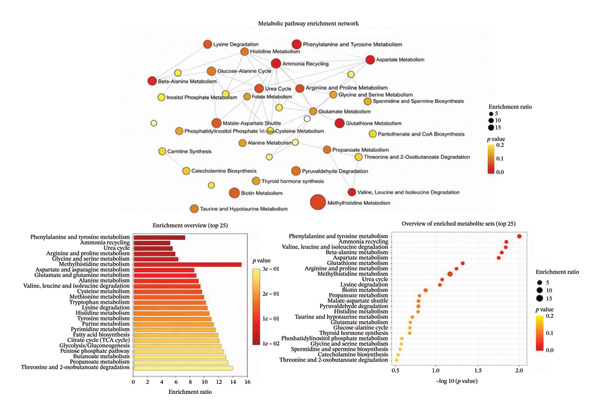
Pathway enrichment analysis overview. Displays metabolic pathway enrichment analysis results with false discovery rate (FDR) correction. Bubble size represents pathway impact (number of metabolites), and colour intensity represents statistical significance. Pathways analysed using MetaboAnalyst 5.0 with topology analysis. Significance threshold: raw *p* < 0.05; FDR values shown in Table 1 for multiple testing correction assessment.

In our previous experiments, we demonstrated that short‐term exposure to blue light (28 days and 12:12 h) significantly altered retinal cells and metabolites [4, 5, 14]. We reported that short‐term exposure to blue and blue‐enriched light diminishes retinal cell density and affects the homocysteine and taurine pathways (Figure 4). Here, we show that distinct metabolomic signatures and changes in their biological roles are associated with controls, LE and BBLs (CP and DB) for prolonged durations in the VF.

We observed alterations in the phenylalanine, tyrosine, valine, leucine and isoleucine metabolism pathways. The inhibition of lysyl oxidase (LOX) through auto‐oxidation of AA activates reactive oxygen species (ROS) [15–17]. The enrichment of the glutathione metabolism pathway (Table 1, FDR = 0.74) suggests the engagement of oxidative stress defence mechanisms. While the FDR value indicates caution in interpretation, the biological plausibility is strong given that blue light generates ROS through photochemical reactions involving retinal chromophores and mitochondrial photosensitizers. The observed alterations in homocysteine‐related metabolites support this interpretation, as homocysteine dysregulation is known to modulate LOX activity and ROS generation in retinal tissues. These changes may be caused by pathways that signal cell death, which are associated with damage to retinal ganglion cells (RGCs) [18].

The functional consequences of these metabolomic alterations for vitreous and retinal health are significant. Glutamate, the retina’s primary excitatory neurotransmitter, showed marked dysregulation in our chronic light‐exposure model. The close biochemical coupling between vitreous and retina means that altered vitreous glutamate levels reflect and potentially exacerbate retinal neurotransmission imbalances. Chronic glutamate receptor overstimulation leads to excitotoxicity through calcium influx and mitochondrial dysfunction, ultimately causing RGC death—a process implicated in glaucoma and other neurodegenerative retinal diseases.

The close connection between the vitreous and retina may be responsible for this phenomenon. Changes in the biochemistry of the vitreous, including alterations in growth factor and other protein levels, can affect the proliferation of neural and glial retinal cells. This, in turn, may impact on the health and function of the retina in LE, significantly reducing the levels of nonessential AAs such as glutamate, aspartate and asparagine. TCA cycle metabolites, such as citrate and pyruvate, were reduced considerably in the chronic LE group, leading to decreased energy production post‐LE; BBLs rescued these metabolites.

The observed reduction in TCA cycle intermediates (citrate and pyruvate) and the altered ATP/ADP ratios indicate compromised energy metabolism, with direct functional implications for the highly metabolically active retinal tissues. Photoreceptors and RGCs are among the most energy‐demanding cells in the body; impaired oxidative phosphorylation capacity compromises phototransduction, signal transmission and cellular maintenance. The partial restoration of these metabolites by BBLs suggests that reducing blue light exposure preserves cellular bioenergetic capacity.

LE significantly increased ADP, and decreased ATP levels were observed after 90 days of cumulative LE, to a greater extent than with BBLs and NC. Significant molecular perturbation was also observed in the VF after 90 days of LE exposure, specifically in terms of the levels of the excitatory AAs like glutamate and alanine rhodopsin. No significant difference in dopamine or acetylcholine levels was observed between the NC and LE groups. However, serotonin levels were abundantly elevated in the LE group compared with the BBL group. These findings suggest that imbalances in neurotransmitters might be triggered in retinal degeneration. Altered glutamate was an important finding in our analysis.

We detected a noticeable difference in neurotransmitter levels in the LE at 90 days. Notably, taurine and hypoxanthine levels were decreased, which is particularly concerning given taurine’s role as a neuroprotector in the retina since shorter wavelengths cause RGC damage [19]. Taurine depletion in the vitreous is particularly concerning from a neuroprotective perspective. As a key retinal osmolyte and antioxidant, taurine protects photoreceptors against oxidative damage, regulates calcium homoeostasis and stabilises membranes. The reduced vitreous taurine levels observed after chronic blue light exposure likely reflect both increased oxidative consumption and potential release from stressed or dying retinal cells, particularly RGCs, which are vulnerable to short‐wavelength phototoxicity. This cascading event could have triggered the taurine mechanism; thus, taurine might have been released from the RGC and become detectable in the vitreous.

Glutamate signalling and homoeostasis are essential for regulating visual signals in the retina. Disruptions can lead to degenerative conditions in retinal pathologies, especially those related to RGC death. It is possible that the downregulation of glutamate in LE is caused by the disruption of RGCs induced by blue light exposure. Long‐term activation of glutamate receptors can cause neurotoxicity, leading to cell death and neuronal dysfunction. As the most abundant AA in the central nervous system, glutamate is a major excitatory neurotransmitter [20]. Ensuring optimal brain function requires maintaining the viability of neurons in the central nervous system. It is imperative to implement effective measures to eliminate excess glutamate, as an overabundance of this neurotransmitter can lead to the overstimulation of specific receptors, such as the N‐methyl‐D‐aspartate and α‐amino‐3‐hydroxy‐5‐methyl‐4‐isoxazolepropionic acid receptors. This, in turn, can lead to excitotoxicity, with detrimental effects on neuronal activity.

During our previous short‐term exposure and current experiment, we observed altered glutamate levels in the vitreous. This could trigger retinal neuronal damage under cumulative light exposure conditions. Cis‐hydroxyproline, a proline analogue, inhibits collagen synthesis and impairs the attachment and migration of bovine RPE cells in a dose‐ and time‐dependent manner. A noteworthy decrease in proline levels was observed in patients with proliferative vitreoretinopathy. This alteration in proline metabolism could indicate a shift towards collagen synthesis rather than proline catabolism. We found that proline degradation in LE and BBLs may indicate decreased collagen synthesis due to cumulative light exposure, thereby affecting vitreous‐bound structures. Altered proline metabolism affects collagen synthesis in the vitreous and the vitreoretinal interface. Changes parallel those seen in proliferative vitreoretinopathy, suggesting potential structural consequences beyond acute metabolic perturbations.

Phenylacetyl glutamine (PG) is a metabolic product of the essential AA phenylalanine that is altered in the plasma and vitreous. An elevated PG can stimulate platelets and increase the likelihood of cardiovascular issues in diabetic patients. We hypothesize that the increase in PG in the vitreous was significantly influenced by the bloodstream. Exposure to blue light during the morning hours can affect glucose, triglyceride and cortisol levels [21], body weight and metabolism and the circadian rhythm. Since phenylalanine is altered in the LE group, it may trigger oxidative stress to the retina, trabecular meshwork and crystalline lens.

Regarding potential circadian influences, phenylalanine metabolism is known to be circadian regulated, and chronic exposure to blue light can disrupt circadian rhythms, with downstream metabolic consequences. While our 12:12 h light–dark cycle maintained basic circadian structure, the blue‐enriched spectrum during light phases may have affected peripheral circadian oscillators in ocular tissues. This could contribute to the observed metabolic alterations beyond direct phototoxic mechanisms.

Our findings align with established mechanisms of blue light‐induced retinal damage through oxidative stress pathways. Previous studies have demonstrated that exposure to short‐wavelength light increases mitochondrial ROS production, lipid peroxidation and protein oxidation in retinal pigment epithelium and photoreceptors. The metabolomic signature we observe, particularly AA dysregulation, altered energy metabolism and engagement of the antioxidant pathway is consistent with chronic oxidative burden.

Exposure to bright light can cause damage to the VF, leading to symptoms such as floaters, flashes of light and blurred vision. Studies have demonstrated a significant correlation between biochemical modifications in the vitreous and those in the retina [22], which could provide valuable information about pathological changes in the retina. There are well‐documented studies on the effects of blue light exposure on retinal damage [23–28]. In addition, altered levels of vitreous metabolites are associated with increased apoptosis in light‐induced retinas [27, 29–31].

There are no documented studies on the effects of chronic exposure to blue light on vitreous metabolites. We observed altered metabolites of shorter and longer duration, partially protected by commercially available BBLs; these metabolomic fluctuations may alter the intraocular environment and cause damage to bound vitreous tissues.

While we implemented rigorous controls over environmental, feeding and handling variables, we acknowledge that eliminating all confounding factors is inherently challenging in chronic exposure models. Subtle differences in individual stress responses, circadian phase variations despite synchronised sampling or systemic metabolic fluctuations related to feeding patterns may contribute to minor variance in metabolomic profiles. However, the consistent directional changes observed across multiple related metabolites within enriched pathways, coupled with partial restoration by BBLs, suggest that the primary signals reflect genuine light‐exposure effects rather than confounding variables.

The differences observed in this study may not accurately reflect the precise levels of neuromodulators present in the vitreous and retina, as the levels of chemical compounds can influence them. Furthermore, the present study examined only aggregate metabolites, which may not provide comprehensive information about specific metabolites and their interactions. It is crucial to consider these limitations when interpreting the study’s findings. Further research and testing may be necessary to confirm these findings and evaluate the effectiveness of different types of blue light blocking.

## 5. Conclusion

To determine the influence of light exposure, we compiled a summary of the changes in metabolic differences within the vitreous. Our findings suggest that persistent exposure to cumulative blue light can alter vitreous metabolites, potentially contributing to damage to retinal neurons and vitreous tissues. The two BBLs we tested may significantly affect the replenishment of vitreous metabolites after prolonged exposure to light.

## Author Contributions

Conceptualization, Nagarajan Theruveethi, Manna Valiathan, Manjunath B. Joshi, Radhika R. P., Shailaja Bhat and Shama Prasada Kabekkodu; methodology, Nagarajan Theruveethi, Manna Valiathan, Manjunath B. Joshi, Radhika R. P., Shailaja Bhat and Shama Prasada Kabekkodu; software, Nagarajan Theruveethi and Manjunath B. Joshi; validation, Nagarajan Theruveethi, Manna Valiathan, Manjunath B. Joshi and Shama Prasada Kabekkodu; formal analysis, Nagarajan Theruveethi and Manjunath B. Joshi; investigation, Nagarajan Theruveethi, Manna Valiathan, Manjunath B. Joshi, Radhika R. P., Shailaja Bhat and Shama Prasada Kabekkodu; resources, Nagarajan Theruveethi, Manna Valiathan, Shailaja Bhat and Manjunath B. Joshi; data curation, Nagarajan Theruveethi, Manna Valiathan, Manjunath B. Joshi, Shailaja Bhat and Shama Prasada Kabekkodu; writing–original draft preparation, Nagarajan Theruveethi, Manjunath B. Joshi, Radhika R. P. and Shama Prasada Kabekkodu; writing–review and editing, Manna Valiathan, Manjunath B. Joshi, Shama Prasada Kabekkodu and Shailaja Bhat.

## Funding

This research was funded by the Science & Engineering Research Board (SERB) (a statutory body of the Department of Science & Technology, Government of India), grant number EMR/2017/004341.

## Consent

The authors have nothing to report.

## Conflicts of Interest

The authors declare no conflicts of interest.

## Data Availability

The data presented in this study are available on request from the corresponding author.
